# An Improved Comprehensive Medication Review Process to Assess Healthcare Outcomes in a Rural Independent Community Pharmacy

**DOI:** 10.3390/pharmacy7020066

**Published:** 2019-06-17

**Authors:** Geoffrey Twigg, Tosin David, Joshua Taylor

**Affiliations:** 1 Apple Discount Drugs, Salisbury, MD 21804, USA; 2 School of Pharmacy, University of Maryland Eastern Shore, Princess Anne, MD 21853, USA; tdavid@umes.edu (T.D.); jtaylor4@umes.edu (J.T.)

**Keywords:** adverse drug events, brown bag, pharmacy, medication reconciliation, pharmacy clinical services

## Abstract

For years many pharmacists have been performing ‘brown bag’ medication reviews for patients. While most pharmacists and student pharmacists are familiar with this process, it is important to determine the value patients receive from this service. Over the course of this study the authors attempted to modernize the medication reconciliation process and collect data on patient prescription drug and over-the-counter drug use, along with quantifying the types of interventions the pharmacy’s clinical staff performed for patients during this process. The pharmacy partnered with a Quality Improvement Organization to trial their Blue Bag Intervention (BBI) program. The BBI program offered several additional services to the traditional brown bag review. The BBI was instituted as a follow-up tool in the pharmacy’s diabetes self-management education/training clinic to aid in patient follow-up and help the clinical staff identify medication-related events such as medication adherence issues and drug–drug interactions. The clinical staff identified approximately 2.2 events per patient with over 50% being issues that affected patient safety.

## 1. Introduction

There are myriad potential clinical, humanistic, and pharmacoeconomic outcomes that a patient may experience as a result of medication use. Healthcare practitioners utilize medications to improve a patient’s medical condition(s); however, adverse events, ranging from minor adverse drug events (ADEs) to patient death, may occur. Medication use, including both prescription and over-the-counter (OTC) drugs, are common among older patients as well as those with complex and multiple medical conditions. Polypharmacy, the concurrent use of multiple medications by the same patient at different pharmacies, can place individuals at an increased risk for ADEs [1]. Building in a mechanism for pharmacists to prevent and address these adverse events can lead to improved healthcare outcomes while decreasing healthcare expenditure.

In a recent study of hospital discharges, patients who had discrepancies in their medication reconciliation were twice as likely to experience a readmission within 30 days of discharge compared to patients who had had an accurate medication reconciliation performed. In addition, a secondary analysis of the data utilizing pharmacists’ medication reviews and patient interviews showed that 89% of patients had at least one potential adverse drug event (pADE) [2]. Another study looked at the role of incorporating a non-dispensing pharmacist into general practice. This study showed the impact that a pharmacist-delivered medication reconciliation could have regarding tailored solutions for individual patients, relieving interdisciplinary tensions of overlapping tasks, and the integration of more quality metrics into medication management [3]. Within a community pharmacy, pharmacists conducted medication reviews to determine medication-related issues. However, this was not conducted in a prospective way, such as by incorporating a patient interview with the medication review [4].

One successful strategy for reducing ADEs is to engage the patient in a comprehensive medication review process with medication reconciliation, often referred to as a ‘brown bag’ medication review. During a ‘brown bag’ medication review, patients will place all their medications, prescribed and taken OTC, in a bag and bring them into a medical appointment with a healthcare professional.

Studies involving a ‘brown bag’ medication review have used various methodologies and designs to determine patient medications, best practices, settings, outcomes, and effectiveness measures. Many reviews included only prescription medications versus reviews that include additional OTC drugs, vitamins, and herbal supplements. Another discrepancy arises from the fact that many reviews did not define medication- or drug-related problems in the same manner [5].

It is estimated that 93% of the American population lives within 5 miles of a community pharmacy and on average a patient will see their pharmacist 35 times a year [6]. This makes the pharmacist one of the most accessible healthcare professionals available to the patient. Pharmacists, as a result of their training, are in a unique position to help patients understand their medication regimen along with the cause and effect between the medication(s) a patient takes and their intended or unintended effect(s).

The authors hypothesize that a structured, sustainable medication reconciliation program that partners patients with a pharmacist with whom they have an established relationship and features ‘built-in’ medication safety measures will result in improved patient health outcomes. The aim of this study was to define and classify medication therapy problems identified by a clinical pharmacist in a community pharmacy setting. Moreover, the results may enable pharmacies to show the value of their involvement to third-party insurance payers in order to justify expanding the role of pharmacist billable services.

Numerous examples showing the impact of pharmacist-driven medication reconciliation programs on improving health outcomes can be found in the literature [7,8,9,10,11]. The majority of studies involving medication reconciliation programs have used multiple providers and varying program settings. Previous studies tended to report the findings of ADEs in a retrospective manner, such as interventions found during a chart review [12,13]. This study aimed to show a prospective approach to medication reconciliation with pre-identified health outcome-related events during the patient’s medication review. This study also sought to show the value of utilizing pharmacists practicing in a community pharmacy setting.

## 2. Methods/Intervention

Apple Discount Drugs is a large, independent community pharmacy that has been operating on the lower eastern shore of Maryland since 1971. Apple Discount Drugs’ pharmacy group consists of four community pharmacy locations, a closed-door home infusion pharmacy, and Core Clinical Care—a separate company that houses the pharmacy’s clinical programs. The pharmacy runs a diabetes center and it is accredited by the American Association of Diabetes Educators. It is the only pharmacy-based accredited diabetes center in the area and one of just a few such centers nationwide. The pharmacy also provides extensive medication therapy management (MTM) services [14].

The objective of this study was to implement a structured pharmacist-driven comprehensive medication review program and to identify both actual and potential ADEs among rural patients referred to the pharmacy for diabetes self-management education and training (DSME/T) or for comprehensive medication management. The medication-related problems that were identified by a pharmacist were then categorized according to the type and severity of the event. Medication-related problems included issues involving a possible risk to patient safety, issues surrounding medication adherence, communication errors between the patient and prescriber, and duplicate medications. This study was also designed to set a groundwork for future studies to determine the relationship between proactive pharmacist intervention and specific patient health outcomes such as effects on blood pressure and hemoglobin A1C. The pharmacy used the Blue Bag Initiative (BBI) program, developed by their Quality Improvement Organization (QIO), to provide a structured platform for the pharmacy to conduct medication reconciliation and perform comprehensive medication reviews. The BBI also assisted the pharmacy in capturing pharmacy-related outcomes related to pharmacist-led clinical services.

The BBI intervention differs from the traditional ‘brown bag’ interview in a number of ways. First, the BBI includes a data collection tool for pharmacists to record the number and types of ADEs, pADEs, and interventions that were identified during the medication review (Table 1). The BBI also ensures patient engagement by creating the expectation that the medication reconciliation process is an ongoing activity—for instance, a reusable, blue drawstring medication bag and a patient appointment card were provided to patients. The patient would be able to keep all medications in the medication bag for easy transport to medical appointments or for safekeeping. The BBI allows for medications that are active on the patient’s regimen to be separated from expired and discontinued medications by including a separate white plastic bag.

The study was open to patients who had been referred to the pharmacy’s 10 (DSME/T), past graduates of the program, and patients that were referred to the pharmacy’s MTM program for a comprehensive medication review (CMR). Patients were excluded if they were only referred to the pharmacy for a targeted medication review.

The diabetes education classes were taught by a pharmacist with a certified diabetes educator credential. The prescriber could refer the patient for group classes, or if the patient had special needs the prescriber could elect to have the classes taught one-on-one. The diabetes education cycle began with a one-on-one meeting so the pharmacist could gauge the patient’s understanding of their disease, followed by 3 h classes to teach the fundamentals of managing diabetes. A patient’s third-party payer benefits for follow-up upon completion varied according to the payer. Many of the commercial payers that the pharmacy contracts will allow for diabetes education follow-up on an as needed basis according to the patient’s needs. Patients that have Medicare benefits are allowed 2 h of DSME/T follow-up for each subsequent year following the completion of the class cycle. This lag time in benefits, in many cases, can cause a care gap as many patients need medication management in the months following the successful completion of a DSME/T class cycle, when the patient is able to begin making lifestyle modifications.

The intervention was overseen by a pharmacist from the clinical team at Apple Discount Drugs. The clinical team consisted of two pharmacists who also held Certified Diabetes Education credentials and a licensed pharmacist completing a Community Pharmacy Residency program during their first postgraduate year.

Reviews were standardized to the BBI to maintain a uniform distribution of pharmacy care. Once the participants agreed to take part in the intervention, the pharmacist contacted their primary care provider to send an updated medication list, progress notes, and requests for intervention when necessary. Participants were instructed to put all their medications (including OTC medications, vitamins, herbal supplements, and eye drops) into the blue bag and to bring it with them to the pharmacy for their scheduled appointment. Apple Discount Drugs invited DSME/T class participants referred to the pharmacy to sign up for the BBI intervention as an extra benefit to attending the class sequence. Participants could choose to opt out of the Blue Bag comprehensive medication review with a pharmacist and still take part in the DSME/T classes.

The pharmacist reviewed the medications at the conclusion of the interview, created an updated medication list incorporating any medication changes, recorded patient outcome data into the QIO’s BBI collection tool, and discussed the results with study participants. The pharmacist would also, when necessary, secure outdated, discontinued, or contraindicated medications and instruct the patient on how to safely discard medications.

## 3. Results

There were 110 patients who were offered the opportunity to participate and 73 patients agreed to take part in the intervention (Table 2). Patients who were referred for targeted medication reviews or patients who received telephonic MTM were excluded from participation. Over 50% of the patients in this study were Medicare beneficiaries. There was an average of 8.5 medications (prescription and OTC) per patient, with higher averages of 11 and 11.8 medications per patient in the age ranges of 86–90 years and 41–45 years, respectively. There was a similar number of medications per patient seen amongst men (average 8.4) and women (average 8.6). For patient-identified ethnic groups, there was an average of 7.6 medications per patient amongst Blacks/African-Americans and an average of 8.2 amongst Whites/Caucasians. The majority of patients seen were being treated for metabolic syndrome. Of the patients taking part, 91.8% were treated for hypertension, 87.7% were treated for diabetes, and 63% were treated for hypercholesterolemia (Table 3).

The majority of patients reported bringing all of their medications to their appointments with the pharmacist (87.7%). Participants could also state what condition their medications were prescribed for (82.2%). Less than half (49.3%) stated that any healthcare practitioner had inquired about their medication list in the past 6 months (Table 4).

**Figure 1:**
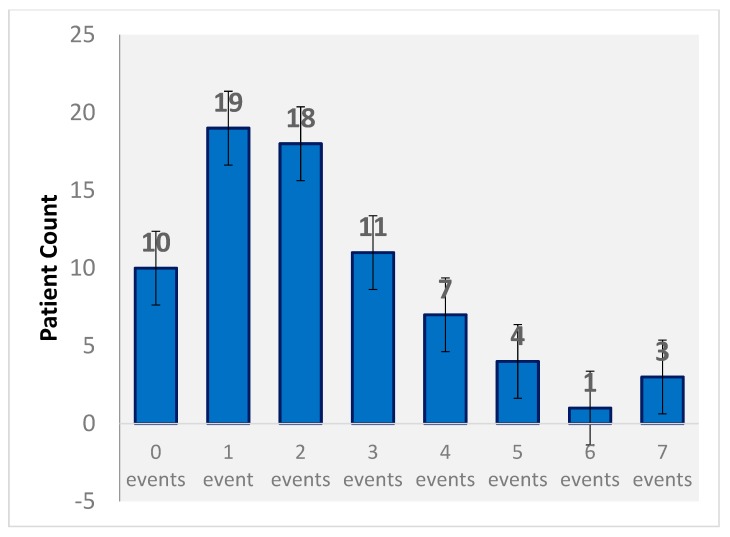
Events per patient.

Pharmacists identified potential and actual ADEs utilizing the Blue Bag Initiative (Table 5). There was an average of 2.2 identified events per patient, with the highest number of ADEs (7 identified events) found in three patients (Figure 1). Over 50% of pADEs identified were related to a possible harm in patient safety. A 16% correlation was seen between an increased number of medications per patient and the number of identified adverse drug events. A weaker correlation of 9% was seen between the number of identified conditions per patient and the number of identified adverse drug events.

Out of the 674 medications reviewed, 162 were over-the-counter medications (24% of the total medications reviewed). This was significant as many patients did not consider an OTC medication to be part of their medication list and many did not inform their prescribers of OTC medication use. There was a 23% correlation between patients who had OTC medications and an event occurring.

## 4. Limitations

This study was limited to the small number of patients that were referred to the pharmacy and agreed to participate. The pharmacists performing the medication reviews relied on the patient for an accurate accounting of medications added to the Blue Bag. The pADEs that were documented by the pharmacists were subjective, especially when considering what constitutes a risk to patient safety, which was the most common type of pADE reported. There is still a large variation in the literature as to how these events are defined. The pharmacists participating in this study determined that a risk to patient safety would include medication-related effects of the patient’s regimen outside of taking one specific medication. Despite this initial training, the reporting of pADEs might have varied from reviewer to reviewer. This means that training, implementation support, and periodic data collection check-ins are recommended.

The low sample size prevented further assessment of the intervention’s impact on hospital utilization, such as ED visits, admissions, readmissions, and observational stays. A systematic review published in 2017 notes the importance of measuring factors such as medication therapy management and patient-specific variables [15]. A further study investigating the effects of this intervention on the abovementioned outcomes would be a recommended expansion of this initial study.

## 5. Discussion

Patient empowerment helped the investigators drive this study. Patients reported a better understanding of the reasons why they were taking specific medications and how those medications worked. This allowed patients to relate side effects to a particular medication, and empowered them to become more active participants in their healthcare. Research has shown that as patient empowerment is improved there are increased levels of patient involvement and better patient behaviors such as medication adherence [16].

Polypharmacy increases with the number of chronic conditions and prescribers. As the number of medications goes up, so does the potential for medication-related problems. This is especially true in older patient populations. A recent study performed in a geriatric patient population showed that patients receiving a mean of 10 medications had the incidence of medication-related problems drop from 86.6% to 56% when a pharmacist was involved in the medication reconciliation process [17]. In this study the need for medication reconciliation was shown when surveying the individuals, as slightly less than half had not had their medications reviewed in the last 6 months. Retrospective research has shown the risk of ADEs in ambulatory settings. Patients taking multiple medications, such as non-opioid analgesics, anticoagulants, diuretics, and anti-seizure medications, have been shown to be at an increased risk [18]. This was further exacerbated by the pharmacist finding 2.2 drug-related issues per medication review. Majority of the participants that completed this study had an understanding of what their medications were for. Conversely, a third of the of patients were not taking their medications as prescribed, ranking as the second-most identified event during the medication review. The investigators also noted that many patients did not understand the associated side effects of medications. After the interview with the pharmacist utilizing the Blue Bag review, patients were able to identify the causes and effects of medication use. Cahn et al. looked at the recognition of drug-related problems before and after pharmacist intervention. There was a significant increase in identified problems seen when a pharmacist reviewed the patient’s medications, resulting in increased clinical and compliance interventions [19].

There are a number of practical and innovative ways the BBI can assist in improving and quantifying pharmacy workflow processes, many of which directly impact patient safety measures. The BBI provides pharmacists with a mechanism to remove outdated and discontinued medications from a patient’s medication regimen. Due to the expense of medications, often times a patient would hesitate to discard a medication once it had been discontinued. The patient would want to have the medication on hand in the event that the prescriber would restart the medication, so as to avoid an additional copayment. Having a discontinued medication stored with other medications resulted in patient confusion and a potential patient safety issue. Separating these medications from active medications proved to be a valuable intervention to patient safety.

Studies have been published on medication discrepancies during medication reconciliation and the effects of this on patients. In this study, the pharmacist found instances where the patient reported medications of which the prescriber was unaware [20]. The likelihood of this occurring became more common as the number of prescribers a patient saw increased. Each prescriber performed their version of medication reconciliation at their practice site. Quite often, there would be discrepancies among the medications a patient was prescribed from practice to practice. The BBI intervention gave the pharmacist the ability to communicate current medications and medication changes to all of the patient’s prescribers as well as alert prescribers to difficulties that a patient may experience between visits.

There was a correlation between OTC medications and pADEs identified within the study; this afforded the pharmacist with an opportunity to improve patient care. Many patients felt that OTC medications were safe and effective for use without seeking treatment by a health professional. This can be complicated by a patient’s use of other nonprescription and prescription medications, especially as the amount of either increases. Pharmacists have an opportunity to encounter the patient and review their medications to assess the harm of starting or stopping a nonprescription product and reporting this information to other healthcare professionals.

As patients continue managing their various condition(s), their medication dose needs to be considered for safety and efficacy. Often, medications need to be tapered up and titrated down. Pharmacist are in a unique position to interview the patient and devise an appropriate medication plan for the patient by utilizing the Pharmacists’ Patient Care Process (JCPP) [21]. Pharmacists utilize evidence-based medicine and patient-specific parameters to determine an appropriate medication dose and frequency for a patient. Pharmacists play an integral role in providing patient counseling on appropriate medication administration. This encompasses education not only on a specific medication, but the patient’s entire medication regimen. Pharmacist guiding patients on various interactions with supplements, herbal medications, and OTC products to avoid while taking their prescribed medication regimen can circumvent potential adverse drug events. During the interview process, many patients initially did not consider OTC and natural products to be a part of their medication regimen. It was not until the pharmacist asked pointed questions or explained how OTC products could impact a patient’s overall health that many patients understood the importance of complete and accurate medication reconciliation. Several patients later reported taking their blue bags and medication cards to doctor’s appointments with primary and specialty care. The BBI also helped patients to understand the importance of communication with prescribers to alert them to changes to their medication regimen.

Another area where the BBI can assist a pharmacy is in providing the pharmacy with a mechanism to quantify interventions in a systematic format in order to track and share intervention data with other healthcare professionals and insurers [22]. Many pharmacies that provide clinical services to patients perform similar types of interventions as those noted by the study investigators. A study by Tetuan et al. utilized a system where recently discharged inpatients were able to utilize the pharmacy’s service in order to identify potential drug-related problems. By utilizing the BBI initiative as a marker for cost avoidance, future studies on the resolution of drug-related problems will assist pharmacies in sustaining initiatives with local health systems [23]. Without the ability to show the value of these services to third-party payers, these pharmacist interventions become value-added services. An intervention utilizing the BBI intervention showcased an average of $218 to $319 in potential savings per completed medication review [24]. When pharmacists are able to quantify and assign a value to these services, they are then able to market clinical interventions in the community pharmacy setting and, in turn, use these programs to increase pharmacy revenue [25].

## 6. Conclusions

This study highlighted the impact of pharmacist-driven medication reconciliation and reviews. The pharmacy’s implementation of the BBI demonstrated this program to be a viable way to perform medication reconciliation and identify pADEs so as to improve health outcomes for patients. By collecting specific data points from the patient and highlighting areas of correlation, pharmacists will be equipped to display where their skills lie in medication management. Patients embraced the interactive nature of the BBI and seemed to be willing to take a more active role in their healthcare as a result.

Apple Discount Drugs is also expanding the use of the BBI to try to determine the cost savings associated with the prevention of ADEs, and to use the outcomes from the BBI to expand the number of billable services offered to pharmacies in coordination with third-party payers. The expansion of the availability of this medication reconciliation program could have a great impact on the care afforded to patients and could strengthen the implementation and data collection support offered to healthcare providers. Additional studies on healthcare outcomes and control group comparisons are recommended for future impact analyses of pharmacist-driven medication reconciliation in the community pharmacy setting.

## Figures and Tables

**Table 1:** Blue Bag Initiative Event Classification.

Event Type
A possible risk to participant safety
Participant not taking medication as prescribed
Medication was correct, but dose was not
Participant stopped taking prescription meds without telling a clinician
Participant taking a new over-the-counter (OTC) med or supplement without telling a clinician
Drug–drug interactions could be possible
Participant failed to get medication(s) refilled
Expired medications
Participant had contraindication for one or more medications
Participant taking new prescription med (from another doctor) without telling clinician
Pill bottles brought in did not match the medication list in the patient’s medical record
Duplicate medications
Stopped taking an OTC med or supplement without telling a clinician
Participant changed to cheaper medication

**Table 2:** Patient demographics.

Population	N = 73 (100%)
**Sex/Gender**	
Male	46 (63%)
Female	27 (37%)
**Race/Ethnicity**	
African American/Black	8 (11%)
Caucasian/White	46 (63%)
Patient preferred not to disclose	19 (26%)
**Age (years)**	
0–30	0
31–60	17 (23.3%)
61–90	50 (68.5%)
91+	1 (1.4%)
Patient preferred not to disclose	5 (6.8%)

**Table 3:** Conditions/disease indication by patient count.

Condition/Disease Indication	Patient Count	% of Total Patients
Hypertension	67	91.8%
Diabetes	64	87.7%
Cholesterol	46	63.0%
Pain	29	39.7%
Allergies	24	32.9%
GERD	19	26.0%
Depression	16	21.9%
Edema	10	13.7%
Anxiety	9	12.3%
Neuropathy	7	9.6%
Gout	5	6.8%

**Table 4:** Patient medication reconciliation survey responses.

	Yes	No	No Response/Unsure
Did the participant say they brought in all their medications?	64 (87.7%)	8 (11%)	1 (1.3%)
Has anyone asked about the participant’s medications in the last 6 months, not including today’s discussion?	36 (49.3%)	36 (49.3%)	1 (1.3%)
Could the patient state what each medication was for?	60 (82.2%)	9 (12.3%)	4 (5.5%)

**Table 5:** Number of medication-related events identified.

Event Type	Patient Count	Percent
A possible risk to participant safety	41	56.20%
Participant not taking medication as prescribed	23	31.50%
Medication was correct, but dose was not	19	26.00%
Participant stopped taking prescription meds without telling a clinician	16	21.90%
Participant taking a new over-the-counter (OTC) med or supplement without telling a clinician	13	17.80%
Drug–drug interactions could be possible	12	16.40%
Participant failed to get medication(s) refilled	12	16.40%
Expired medications	11	15.10%
Participant had contraindication for one or more medications	3	4.10%
Participant taking new prescription med (from another doctor) without telling clinician	3	4.10%
Pill bottles brought in did not match the medication list in the patient’s medical record	3	4.10%
Duplicate medications	2	2.70%
Stopped taking an OTC med or supplement without telling a clinician	2	2.70%
Participant changed to cheaper medication	2	2.70%
